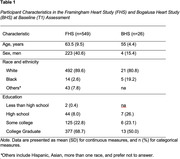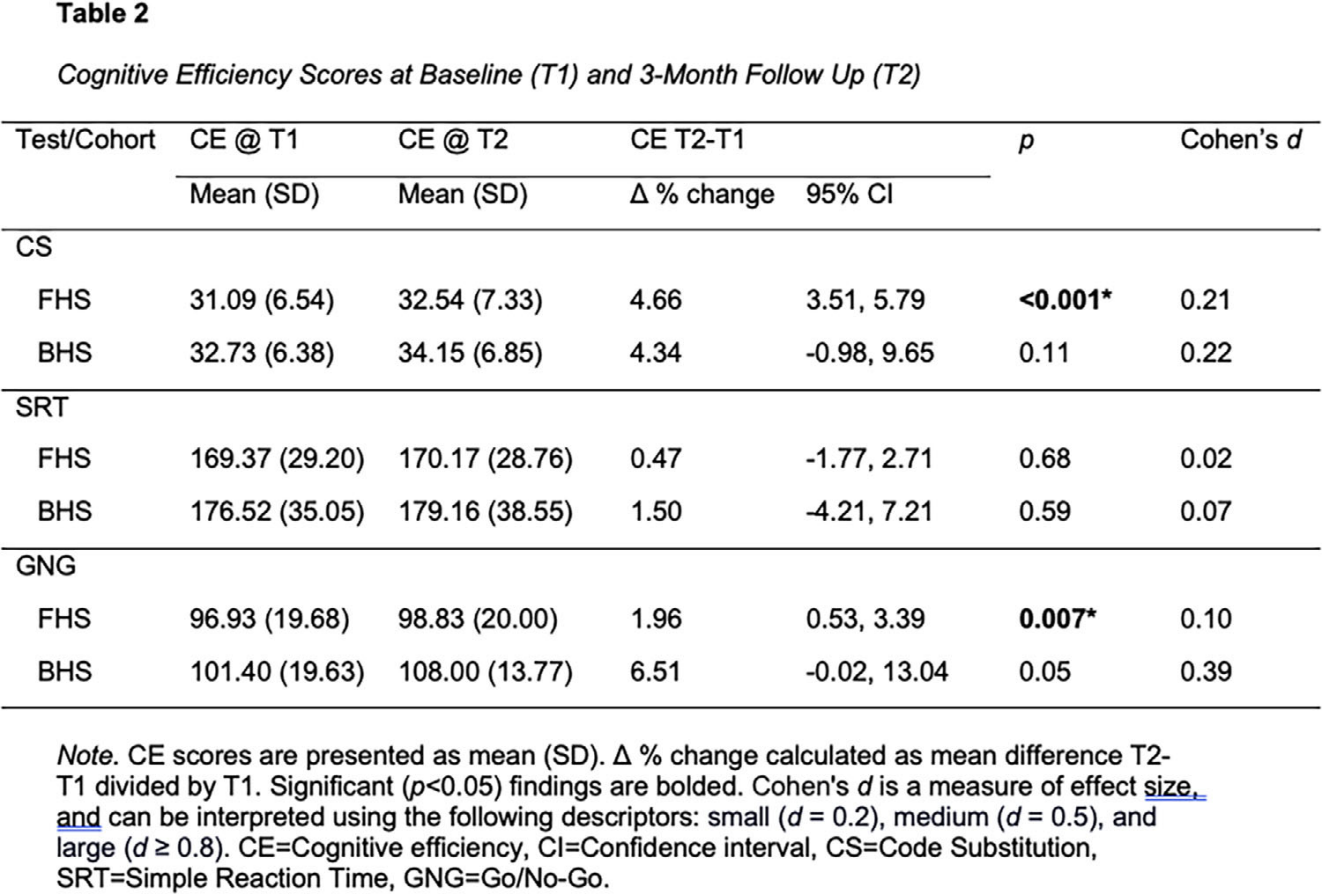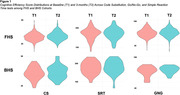# Investigating test‐specific practice effects on self‐administered mobile‐based cognitive tasks in two diverse community‐based cohorts

**DOI:** 10.1002/alz.092876

**Published:** 2025-01-03

**Authors:** Kieffer Christianson, Ileana De Anda‐Duran, Cody Karjadi, Lindsay Hathaway, Jose R Monteverde, Sherral A. Devine, Ting Fang Alvin Ang, Phillip H Hwang, Lydia Bazzano, Vijaya B. Kolachalama, Honghuang Lin, Rhoda Au, Preeti Sunderaraman

**Affiliations:** ^1^ University of Nevada, Reno, Reno, NV USA; ^2^ Boston University Chobanian & Avedisian School of Medicine, Boston, MA USA; ^3^ Tulane University School of Public Health and Tropical Medicine, New Orleans, LA USA; ^4^ Boston University School of Public Heatlh, Boston, MA USA; ^5^ Boston University, Boston, MA USA; ^6^ University of Massachusetts Chan Medical School, Worcester, MA USA; ^7^ Boston University School of Public Health, Boston, MA USA

## Abstract

**Background:**

Repeated self‐administered mobile‐based cognitive assessments are increasingly being utilized to identify preclinical cognitive decline. Repetition of cognitive tests during a short time interval often leads to improved performance (i.e., practice effects) due to familiarity with test structure. Therefore, test‐specific practice effects may delay the detection of subtle decline. Alternatively, the lack of practice effect may indicate a decline in cognitive function. This study investigated whether practice effects were present on three digital tests repeated over a 3‐month interval in two cognitively unimpaired cohorts.

**Method:**

549 cognitively intact participants (average age 63.5 years, 40.6% male, 89.6% white) enrolled in the Framingham Heart Study and 26 cognitively unimpaired participants (average age 55 years, 15.4% male, 80.8% white) from the Bogalusa Heart Study completed three self‐administered subtests from Digital Automated Neurobehavioral Assessment (DANA) on their own mobile device at baseline (T1) and three months later (T2). Cognitive scores were calculated using Cognitive Efficiency (CE), a derived measure of speed and accuracy, for Code Substitution (CS), Simple Reaction Time (SRT), and Go/No‐Go (GNG). CS assesses visual scanning and attention, learning, and immediate recall; SRT measures pure reaction time; and GNG assesses sustained attention and impulsivity. For each cohort, separate paired sample t‐tests examined mean differences in CE between timepoints for all three tests.

**Result:**

On all three tests, similar trends in test performance were found in both cohorts. FHS participant’s CE scores significantly improved at T2 compared to T1 on CS and GNG, but not on SRT (Table 2). Similar trends were observed among BHS participants, with more relative improvements on CS and GNG compared to SRT.

**Conclusion:**

This study found similar trends in test‐specific practice effects on mobile‐based cognitive tests in two different cohorts. CS and GNG are inherently more complex than SRT, which may explain why test familiarity improved performance three months after baseline. This is congruent with traditional paper‐and‐pencil neuropsychological tests, wherein tests with complex stimuli show more practice effects relative to simple tasks. Additional research is needed to determine which tests are more sensitive to detecting earliest indicators of cognitive decline, and whether the presence/absence of practice effects itself are clinically meaningful.